# Obsessive-compulsive symptoms and traits in patients with burning mouth syndrome: a cross-sectional multicentric analysis

**DOI:** 10.1007/s00784-025-06293-6

**Published:** 2025-04-05

**Authors:** Luca Pellegrini, Federica Canfora, Giulia Ottaviani, Cristina D’Antonio, Katia Rupel, Michele Davide Mignogna, Matteo Biasotto, Amerigo Giudice, Gennaro Musella, Vito Carlo Alberto Caponio, Gianrico Spagnuolo, Carlo Rengo, Giuseppe Pecoraro, Massimo Aria, Luca D’Aniello, Umberto Albert, Daniela Adamo

**Affiliations:** 1 Department of Medicine, Surgery and Health Sciences, University of Trieste, Trieste, Italy; 2 Department of Mental Health, Psychiatric Clinic, Azienda Sanitaria Universitaria Giuliano- Isontina– ASUGI, Trieste, Italy; 3 School of Life and Medical Sciences, University of Hertfordshire, Hatfield, UK; 4 Centre for Neuropsychopharmacology and Psychedelic Research, Hammersmith Hospital Campus, Imperial College, London, UK; 5 Department of Neuroscience, Reproductive Sciences and Dentistry, University of Naples “Federico II”, 5 Via Pansini, Naples, 80131 Italy; 6 School of Dentistry, Department of Health Sciences, Magna Graecia University of Catanzaro, Catanzaro, 88100 Italy; 7 Department of Clinical and Experimental Medicine, University of Foggia, Foggia, 71122 Italy; 8 Department of Economics and Statistics, University of Naples “Federico II”, Naples, Italy; 9 Department of Social Sciences, University of Naples “Federico II”, Naples, Italy; 10 Department of Life Science, Health, and Health Professions, Link Campus University, Rome, Italy

**Keywords:** Burning mouth syndrome, Obsessive-compulsive disorder, Obsessive-Compulsive personality disorder, Compulsive personality traits, OCI-R, CPAS

## Abstract

**Objective:**

This study investigates the frequency and characteristics of obsessive-compulsive (OC) symptoms and Obsessive-Compulsive Personality Disorder (OCPD) in patients with Burning Mouth Syndrome (BMS).

**Background:**

Obsessive-Compulsive Disorder (OCD) is a chronic condition involving intrusive thoughts (obsessions) and repetitive behaviors (compulsions), while Obsessive-Compulsive Personality Disorder (OCPD) is a personality disorder characterized by specific traits such as perfectionism, rigidity and need for control. Both conditions frequently overlap, but their prevalence in patients with BMS has never been explored.

**Materials and methods:**

A total of 151 BMS patients were assessed using the Obsessive-Compulsive Inventory-Revised (OCI-R), Compulsive Personality Assessment Scale (CPAS), Visual Analog Scale (VAS), Short-Form McGill Pain Questionnaire (SF-MPQ), Hamilton Anxiety and Depression scales (HAM-A, HAM-D), Pittsburgh Sleep Quality Index (PSQI), and Epworth Sleepiness Scale (ESS). Patients were grouped based on OCI and CPAS scores.

**Results:**

*n* = 123 (81.6%) of our sample were females, with a mean age of 63.19 ± 12.2 years. Clinically significant OC symptoms (OCI-*R* > 21) were observed in 41.7% of the sample, while 37% met OCPD criteria; both OC symptoms and OCPD were present in 24.5% of patients.

**Conclusions:**

BMS patients show a high prevalence of OC symptoms and OCPD traits, which should be taken into account by clinicians and considered in the therapeutic approach, given that they could complicate symptom management.

**Clinical relevance:**

: By identifying these symptoms and traits through OCI-R and CPAS, clinicians may improve treatment strategies, in the perspective of a multidisciplinary tailored and personalized approach.

**Supplementary Information:**

The online version contains supplementary material available at 10.1007/s00784-025-06293-6.

## Introduction

Burning Mouth Syndrome (BMS) is a chronic neuropathic pain disorder characterized by a persistent burning sensation in the oral cavity, often without any identifiable local or systemic cause [1]. This condition is associated with a range of intraoral symptoms, including xerostomia, sialorrhea, dysgeusia, intraoral foreign body sensation, globus sensation, tingling, and itching [2, 3]. Despite the lack of observable physical abnormalities, BMS significantly impacts patients’ quality of life, with symptoms often persisting over long periods [4].

The prevalence of BMS is estimated at 1.73% in the general population, with higher rates observed in older adults and among dental patients, particularly females over 50 years of age [5]. Despite extensive research, the pathophysiology of BMS remains poorly understood. Current evidence suggests that BMS is a multifactorial disorder involving both peripheral and central nervous system dysfunctions [6]. Peripheral neuropathy, particularly affecting small nerve fibers in the oral mucosa, has been observed in some patients [7]. Concurrently, central mechanisms, such as altered pain processing in the brain, are increasingly implicated [8]. Neuroimaging studies have revealed abnormalities in brain regions related to pain perception and emotional regulation, including the prefrontal cortex, insula, and thalamus [9]. This supports the notion that BMS is not solely a sensory disorder but is also intimately connected to emotional and psychological factors [10].

Psychiatric comorbidities such as anxiety, depression, sleep disturbances, and stress are well-documented in BMS patients and are believed to contribute to the chronicity and severity of symptoms [11]. These psychological factors may exacerbate pain perception through dysregulation of the brain’s pain-processing systems, leading to heightened pain sensitivity and poorer treatment outcomes [12].

Despite the known link between emotional distress and chronic pain, specific psychiatric disorders such as Obsessive-Compulsive Disorder (OCD) and Obsessive-Compulsive Personality Disorder (OCPD) have received relatively little attention in the context of BMS, representing a significant gap in the literature.

According to the DSM-5, OCD is characterized by two main features: the presence of intrusive, recurrent and persistent thoughts, urges or images that are experienced as intrusive, unwanted, and cause marked anxiety or distress (obsessions); and repetitive behaviors (e.g., hand washing, ordering, checking) or mental acts (e.g., praying, counting, repeating words silently) that the individual feels driven to perform in response to an obsession and according to rules that must be applied rigidly (compulsions) [13]. This disorder, which is often non-responsive to treatments [14] and hence the need for new forms of therapy [15, 16], has been linked to substantial impairments in both functioning and quality of life, can significantly increase suicide risk in individuals suffering from it [17] and has a major global disability burden [18, 19]. The prevalence of OCD in the general population has been estimated to be around 3.5%, with a peak age of onset of 14.5 years [20, 21], being the fourth most common psychiatric disorder in the general population during lifetime [22]. A substantial proportion of the population - estimated at 21% and 28% in the studies by Ruscio et al. [23] and Fineberg et al. [20]- report subthreshold, but clinically significant, obsessive-compulsive symptoms. OCD is the nosological organizer of the new group of disorders named “*obsessive compulsive and related disorders”*, that includes body dysmorphic disorder, hoarding disorder, excoriation (skin-picking) disorder, hair pulling disorder (trichotillomania) and hypochondriasis [14].

In individuals with chronic pain, the rates are significantly higher, ranging from four to six times the general average [24, 25]. For example, patients with fibromyalgia are up to six times more likely to have OCD, with women being approximately five times more susceptible over their lifetime [26]. This marked overlap between chronic pain conditions and obsessive-compulsive traits highlights a complex relationship that influences both pain perception and management [27].

On the other hand, OCPD is defined by a pervasive pattern of preoccupation with orderliness, perfectionism, and need for control, often at the expense of flexibility and efficiency [28, 29]. While OCD involves distress from uncontrollable thoughts and behaviors, OCPD revolves around a rigid need for control and perfectionism, which may interfere with decision-making, relationships, and adaptability [30]. Although OCD and OCPD are distinct disorders, they frequently co-occur, and share traits such as rigid behavior patterns and hypervigilance and their comorbidity is particularly difficult to treat [31]. Both disorders could complicate pain management strategies: the compulsive behaviors, such as excessive checking of the oral area, and the heightened focus on bodily sensations, both typical of OCD [13, 29], may amplify pain perception, contribute to maladaptive coping mechanisms [32] and interfere with treatment efficacy [24]. Similarly, individuals with OCPD, driven by perfectionism and rigidity, may experience increased sensitivity to pain, be less responsive to gradual changes and have poorer clinical outcomes [33–35].

Given the recognized associations between OCD, OCPD, and chronic pain in other conditions like fibromyalgia, chronic migraine, and temporomandibular joint disorders, it is plausible that a similar relationship exists in BMS, though this area remains underexplored. The focus on OCPD traits in patients with BMS is supported by evidence indicating a notable overlap between BMS and OCD/OCPD characteristics. This overlap highlights the potential for OCPD traits to significantly impact the daily functioning and quality of life in BMS patients [36]. Such connections between BMS and psychopathological traits have been underexplored in existing literature, making this study a valuable addition to the field. Moreover, research by de Souza et al. emphasizes the prevalence of major psychiatric disorders in BMS populations, reinforcing the importance of psychopathological evaluations in the comprehensive management of BMS [37].

Moreover, given our clinical experience, from both the perspective of clinical expertise in BMS and clinical expertise in OCD, we notice that that there is a significant overlap between OC symptoms and OCPD traits, from one end, and BMS to the other. Understanding the frequency of clinically significant OC and OCPD on pain perception in BMS could pave the way to more personalized treatment approaches for this complex disorder.

The primary objective of this study was to assess the frequency and characteristics of OC symptoms and OCPD traits in a cohort of BMS patients using validated assessment tools, including the Obsessive-Compulsive Inventory-Revised (OCI-R) and the Compulsive Personality Assessment Scale (CPAS).

The secondary objective was to investigate the differences between BMS patients with and without clinically significant OC symptoms, as well as in those with and without OCPD, across various clinical parameters. These parameters include sociodemographic factors, risk factors, systemic comorbidities, medication use, pain symptomatology, anxiety, depression, and sleep disturbances.

To our knowledge, this is the first study to evaluate the presence of OC symptoms and OCPD traits in patients with BMS, thus providing novel insights that could enhance both the understanding and treatment of this complex condition.

## Materials and methods

### Study design and participants

This observational, cross-sectional study was conducted at the Oral Medicine Department of the University of Naples “Federico II” between January 2022 and January 2024. The study adhered to the ethical guidelines outlined by the World Medical Association Declaration of Helsinki and received ethical approval from the University’s Ethical Committee (Approval Number: 251/19, February 20, 2019). All participants provided written informed consent, and no compensation was offered for participation. The methodology followed the Strengthening the Reporting of Observational Studies in Epidemiology (STROBE) guidelines [38].

### Participants

A total of 200 BMS affected, aged 18 years or older, Italian speaking participants were recruited; they responded to a series of questionnaires, carried out in in-person visits, with the help of a nurse. BMS was diagnosed based on the International Classification of Orofacial Pain (ICOP, 2020) [1], which defines BMS as a condition characterized by intraoral burning or dysesthetic sensations recurring daily for more than two hours over at least three months, without evident causative lesions upon clinical examination. Laboratory tests confirmed normal values for blood count, glucose levels, glycated hemoglobin, serum iron, ferritin, and transferrin. Patients were excluded if they had any systemic diseases or conditions known to cause oral burning, including neurological or metabolic disorders. Other exclusion criteria included inability to comprehend and complete questionnaires, history of psychiatric or organic brain disorders, ongoing treatment with systemic psychotropic drugs, history of alcohol or substance abuse, medications potentially causing oral symptoms, and diagnosis of Obstructive Sleep Apnea Syndrome (OSAS).

Following the screening process, 151 patients met the inclusion and exclusion criteria and were included in the final analysis.

### Data collection and measures

Each participant underwent a thorough intraoral and extraoral examination performed by an oral medicine specialist (DA and FC) and a psychiatric evaluation (LP and GP). Sociodemographic data, including age, gender, BMI, years of education, family situation, and employment status, were collected. Risk factors such as smoking status and alcohol consumption were also documented in order to explore and define the impulsivity profile of participants (patients with OCD often display impulsive behaviors and not uncommonly abuse substances, in particular the legal ones due to their avoidance and fear of doing something prohibited that would make them feel guilty; therefore, smoking and/or alcohol misuse is quite frequent in individuals with OCD). Oral symptoms were evaluated in intensity, diurnal variation (morning, afternoon, evening), and any improvement during meals. Additional information on systemic comorbidities was gathered. The Age-Adjusted Charlson Comorbidity Index (AACCI) [39] was used to assess comorbidities, providing a validated score for predicting 10-year mortality based on a range of comorbid conditions.

### Pain and psychological assessment

A battery of standardized assessments was employed to evaluate pain intensity, pain quality, anxiety, depression, and sleep quality.

Pain Intensity was measured using the Visual Analogue Scale (VAS) [40], a unidimensional tool where patients rated their pain from 0 (no pain) to 10 (worst imaginable pain) along a 10 cm horizontal line.

Pain Quality was assessed using the Short-Form McGill Pain Questionnaire (SF-MPQ) [41], which evaluates sensory, affective, and evaluative dimensions of pain. Each descriptor in the SF-MPQ is rated on a scale from 0 (none) to 3 (severe) was calculated by summing the ratings.

Depression was measured using the Hamilton Depression Rating Scale (HAM-D) [42]. A score greater than 7 indicates impairment, with scores between 7 and 17 suggesting mild depression, 18–24 indicating moderate depression, and scores above 24 reflecting severe depression.

Anxiety was assessed using the Hamilton Anxiety Rating Scale (HAM-A) [43], which measures both somatic and psychic anxiety symptoms. Each item is rated from 0 to 4, with total scores below 17 indicating mild anxiety, 18–24 suggesting moderate anxiety, and scores above 25 indicating moderate to severe anxiety.

Sleep Quality was evaluated using the Pittsburgh Sleep Quality Index (PSQI) [44], a 19-question self-reported survey assessing various dimensions of sleep over the past month. A total score above 5 indicates poor sleep quality. The Epworth Sleepiness Scale (ESS) [45], a self-administered questionnaire, was also used to measure daytime sleepiness. Scores above 10 suggest excessive daytime drowsiness, with scores above 16 indicating more severe conditions like narcolepsy or OSAS.

The Clinical Global Impressions Severity of Illness (CGI-S) [46] scale was applied to evaluate the overall severity of the patient’s condition.

### Assessment of OCD and OCPD

The OCI-R [47] was administered to assess the frequency, characteristics and severity of OC symptomatology in eligible patients. The OCI-R is a widely used, self-administered questionnaire, available in a validated Italian version [48]. It consists of 18 items that evaluate OC symptoms across six distinct subscales/dimensions:

1. Hoarding (e.g., difficulty discarding possessions),
2. Checking (e.g., repeatedly checking things to avoid potential harm),
3. Ordering (e.g., needing to arrange things in a particular order),
4. Mental Neutralizing (e.g., engaging in mental rituals to counter distressing thoughts),
5. Washing (e.g., excessive washing or cleaning due to contamination fears), and.
6. Obsessing (e.g., intrusive and repetitive thoughts causing significant distress).

Each item is rated on a 5-point Likert scale ranging from 0 (“Not at all”) to 4 (“Extremely”), with higher scores indicating greater severity of OC symptoms; a total OCI-R score is calculated by summing all item responses. We defined, by using standardized criteria from previous research [40], the presence of clinically significant obsessive-compulsive symptoms when a patient had a score equal to or above 21 on the total OCI-R (this score is also considered indicative of likely OCD diagnosis) [47]. Additionally, subscale scores are computed by summing the responses corresponding to each of the six dimensions of OCD, offering insight into the specific areas of obsessive-compulsive behavior in individual participants. The OCI-R has demonstrated excellent psychometric properties, including high internal consistency, reliability, and validity, making it an ideal tool for both clinical and research settings [49].

For the assessment of OCPD, the CPAS was used [28]. OCPD, a pattern of rigid thought processes, is distinguished from OCD [50]. The CPAS is a clinician-administered, semi-structured interview designed to assess personality traits consistent with OCPD, as outlined in the Diagnostic and Statistical Manual of Mental Disorders, 5th Edition (DSM-5); an Italian version is available [51].

The CPAS evaluates eight key items, which align with the eight DSM-5 diagnostic criteria, associated with OCPD, including:

1. Preoccupation with details, rules, and lists, to the point that it interferes with task completion,
2. Perfectionism that hampers productivity and the ability to complete tasks,
3. Rigidity and stubbornness in attitudes and behaviors,
4. Reluctance to delegate tasks or work with others unless they adhere strictly to one’s own way of doing things,
5. Excessive conscientiousness and scrupulosity, including concerns with minor details and strict adherence to rules,
6. Workaholic tendencies, with an overemphasis on work and productivity at the expense of leisure and friendships,
7. Miserliness, where money is hoarded for future catastrophes rather than spent on present needs, and.
8. Inability to discard worn-out or worthless objects, even when they have no sentimental or practical value.

Each criterion is rated on a scale from 0 (“Absent”) to 4 (“Very Severe”), with a maximum total score of 32. In accordance with DSM-5 diagnostic guidelines and criteria, we defined the presence of OCPD when individuals scored 3 or higher on at least four of the eight CPAS items [52]. The CPAS has been validated in both clinical and non-clinical populations and has shown good internal reliability (Cronbach’s alpha = 0.75) and discriminative validity. In this study, the CPAS was administered by trained clinicians with at least five years of experience in evaluating personality disorders. This psychometric scale is designed to underscore the severity of compulsive personality traits. Participants were therefore stratified into subgroups basing on the presence of OC symptoms and OCPD traits (according to the OCI-R total and CPAS total scores): patients with clinically significant obsessive-compulsive (OC) symptoms [53] and those without, and patients with OCPD and those without OCPD [44]. The stratification allowed for a more detailed examination of how OCD and OCPD may interact with the clinical manifestation of BMS, as well as how these psychological factors influence the overall burden of the condition. Further analysis was conducted to explore potential associations between OCD/OCPD symptomatology and BMS-related outcomes such as pain intensity, oral symptoms, sleep disturbances, anxiety, depression, and overall quality of life.

This comprehensive approach ensured that both obsessive-compulsive symptoms and personality traits were thoroughly evaluated, offering valuable insights into the psychosomatic dimensions of BMS and contributing to a better understanding of its multifactorial nature.

### Statistical analysis

Statistical analyses were first conducted on the entire cohort of BMS patients (*n* = 151) using descriptive statistics to provide an overview of their socio-demographic and clinical characteristics.

Frequency of obsessive compulsive symptoms and frequency of obsessive-compulsive personality traits in the sample were calculated: patients with an OCI-R score equal to or higher than 21 were classified as having clinically significant OC symptoms [53]; similarly, for OCPD, individuals were considered to meet the criteria for Obsessive-Compulsive Personality Disorder if they scored 3 or higher on at least four of the eight items of the CPAS (DSM-5 diagnostic criteria) [52]; those not meeting these criteria, were considered to not have OCPD.

Subsequently, comparative analyses were performed to assess possible differences between the BMS + clinically significant OC symptoms group and the BMS without clinically significant OC symptoms group, as well as between the BMS + OCPD and the BMS without OCPD groups. Descriptive statistics, including means, standard deviations (SD), medians, and interquartile ranges (IQR), were calculated to examine socio-demographic data, and clinical characteristics across these groups [54].

The normality of the data was assessed using the Shapiro-Wilk test. Differences in proportions were analyzed using Pearson’s chi-square test, and Fisher’s exact test was employed for categorical variables when the expected frequency in any cell was less than five. The non-parametric Mann-Whitney U test was applied to compare median values for continuous variables such as VAS, T-PRI, HAM-A, HAM-D, PSQI, ESS, and CGI-S scores between the groups. To account for multiple comparisons, Bonferroni correction was applied, with significance considered at a corrected p-value < 0.05.

To control for the risk of type I errors due to multiple comparisons, Bonferroni correction was applied, with significance considered at a corrected p-value of < 0.05, ensuring rigorous control over potential false positives in interpreting results.

All statistical analyses were conducted using R software (version 4.4.1), and a significance threshold of *p* ≤ 0.05 was applied unless otherwise noted.

## Results

Table 1 summarizes the sociodemographic characteristics among the sample of the study. Most patients were female (81.6%, *n* = 123), with a mean age of 63.2 ± 12.2 years; these sociodemographic characteristics are in line with clinical manifestations of BMS and with previous research [11]. The average years of education were 10.0 ± 4.1, and the mean BMI was 26.4 ± 4.3, reflecting a population predominantly in the overweight range. Most patients were married (70.2%), while smaller percentages were single (8.6%), divorced (9.9%), or widowed (11.3%). In terms of employment status, 28.4% were employed, 33.1% were housewives, and 30.5% were retired.

**Table 1:** Sociodemographic profile, risk factors, systemic comorbidities, and drug consumption in the 151 BMS patients

Sociodemographic characteristics	Frequency (%)
**Gender**	
Female	123 (81.6)
Male	28 (18.4)
	**Mean ± SD**
Age	63.2 ± 12.2
Education (in years)	10.0 ± 4.1
BMI	26.4 ± 4.3
***Family situation***	***Frequency (%)***
Married	106 (70.2)
Single	13 (8.6)
Divorced	15 (9.9)
Widowed	17 (11.3)
***Employment status***	***Frequency (%)***
Employed	43 (28.5)
Unemployed	12 (7.9)
Housewife	50 (33.1)
Retired	46 (30.5)
***Smoke status***	**Frequency (%)**
Very Light smokers (< 5 cigarettes)	7 (4.6)
Light smokers (5–10 cigarettes)	9 (5.9)
Moderate smokers (10–15 cigarettes)	17 (11.3)
Heavy smokers (> 15 cigarettes)	11 (7.3)
Non-smoker	107 (70.9)
E-cig	3 (1.9)
Heat-not-burn (HNB) tobacco products	4 (2.7)
***Alcohol use***	**Frequency (%)**
Alcohol 1–2 units	11 (7.3)
Alcohol 2–3 units	6 (3.9)
Alcohol >3 units	5 (3.3)
No alcohol	129 (85.4)
***Systemic Comorbidities***	***Frequency (%)***
Yes	134 (88.7)
No	16 (11.3)
	***Median [IQR]***
Age-Adjusted Charlson Comorbidity Index (AACCI)	2 [1–3]
	***Frequency (%)***
***Drugs Consumption***	***Frequency (%)***
Yes	123 (81.4)
No	28 (18.5)

**Abbreviations**: ACCI: Age-Adjusted Charlson Comorbidity Index; BMS: Burning Mouth Syndrome; BMI: Body Mass Index

Regarding smoking habits, 107 (70.9%) of the patients were non-smokers. Alcohol consumption was infrequent, with 85.4% of the patients reporting no alcohol use.

Systemic comorbidities were highly prevalent in this cohort, affecting 134 (88.7%) of the patients.

Table 2 provides a detailed overview of the oral symptoms, pain characteristics, and neuropsychological profiles of the sample. In addition to the burning sensation, patients frequently reported symptoms such as xerostomia (70.2%), dysgeusia (49.7%), and Globus pharyngeus (40.4%). A significant proportion of patients reported subjective changes in tongue morphology (37.1%), while less frequent symptoms included sialorrhea (20.5%), tingling sensations (13.9%), and intraoral foreign body sensation (13.3%).

**Table 2:** Prevalence of oral symptoms and location, pain pattern and analysis of psychological profile in 151 BMS patients

Symptoms	Frequency (%)
Burning	151 (100)
Xerostomia	106 (70.2)
Dysgeusia	75 (49.7)
Globus Pharyngeus	61 (40.4)
Subjective change in Tongue Morphology	56 (37.1)
Sialorrhea	31 (20.5)
Tingling Sensation	21 (13.9)
Intraoral Foreign Body Sensation	20 (13.3)
Itching	12 (7.9)
Oral Dyskinesia	11 (7.28)
Occlusal Dysesthesia	10 (6.6)
Subjective Halitosis	6 (3.9)
Dysosmia	2 (1.3)
***Location of pain/ Burning***	***Frequency (%)***
Tongue	147 (97.4)
Palate	102 (67.5)
Gums	90 (59.6)
Cheeks	87 (57.6)
Lips	83 (54.9)
Floor Of The Mouth	83 (54.9)
Trigone	77 (50.9)
**Pain**	***Median [IQR]***
VAS	10[8–10]
SF-MPQ	12[8.5–18]
***Symptom pattern***	***Frequency (%)***
Worse In The Morning	7 (4.6)
Worse In The Evening	68 (45.0)
No Difference Between	66 (43.7)
Morning and Evening	
Continuous	68 (45.0)
Intermittent	70 (46.4)
Improve when Eating	51 (33.8)
***Psychological Profile***	***Median [IQR]***
Total score of HAM-A	17[14–20]
Total score of HAM-D	17[14–20]
PSQI	8 [6.5–10]
ESS	5[4–7]
Sleep duration (in hours)	6[5–7]
CGI-S	4[4–5]
	**Frequency (%)**
Insomnia onset prior to BMS diagnosis	97 (64.2)
History of previous mood disorder	79 (52.3)

**Abbreviations**: BMS: Burning Mouth Syndrome; CGI-S: Clinical Global Impression Severity of Illness; ESS: Epworth Sleepiness Scale; HAM-A: Hamilton Rating Scales for Anxiety; HAM-D: Hamilton Rating Scales for Depression; PSQI: Pittsburgh Sleep Quality Index, SF-MPQ: Short-Form McGill Pain Questionnaire, VAS: Visual Analogue Scale

Regarding the location of pain or burning, the tongue was the most affected site (97.4%), followed by the palate (67.6%) and gums (59.6%). 102 (67.5%) BMS affected patients reported a burning sensation diffuse to the entire oral mucosa.

Pain severity, as measured by the VAS, had a median score of 10 [IQR: 8–10], indicating severe pain across the cohort. The T-PRI had a median score of 12 [IQR: 8.5–18], further highlighting the significant pain burden experienced by these patients.

In respect to the symptom pattern, 45.0% of patients reported worsening symptoms in the evening, while 46.36% described intermittent pain and 33.8% of patients reported symptom improvement while eating, suggesting a potential modulating effect of oral activities on the perceived burning sensations.

The neuropsychological profile of the patients revealed elevated anxiety and depression scores. The median score on the HAM-A was 17 [IQR: 14–20], and the HAM-D showed a similar median score of 17 [IQR: 14–20], indicating moderate levels of both anxiety and depression. Sleep disturbances were also prevalent, with a median PSQI total score of 8 [IQR: 6.5–10] and a median sleep duration of 6 h [IQR: 5–7]. Notably, 64.2% of patients reported insomnia onset before their BMS diagnosis, and 52.32% had a history of previous mood disorders.

Table 3 provides a detailed analysis of the frequency of OC symptoms and OCPD among 151 patients with BMS, alongside scores on the OCI-R and the CPAS.

**Table 3:** Prevalence of clinically significant OC symptoms and OCPD as measured by OCI-R and CPAS in 151 BMS patients

OCI-*R*		
Items	Median [IQR]	
OCI-r-1	0 [0–2]	
OCI-r-2	2 [0–3]	
OCI-r-3	2 [0–3]	
OCI-r-4	0 [0–0]	
OCI-r-5	0 [0–1]	
OCI-r-6	1 [0–2]	
OCI-r-7	0 [0–2]	
OCI-r-8	0 [0–2]	
OCI-r-9	2 [1–3]	
OCI-r-10	0 [0–0]	
OCI-r-11	0 [0–2]	
OCI-r-12	2 [1–3]	
OCI-r-13	1 [0–2]	
OCI-r-14	0 [0–2]	
OCI-r-15	1 [0–3]	
OCI-r-16	0 [0–0]	
OCI-r-17	0 [0–2]	
OCI-r-18	1 [0–2]	
**OCI-R TOT**	**17 [10–28]**	
Hoarding:		
Item1 + Item7 + Item13	1 [0–5]	
Checking:		
Item2 + Item8 + Item14	3 [1–7]	
Ordering:		
Item3 + Item9 + Item15	5 [2–9]	
Mental Neutralizing:		
Item4 + Item10 + Item16	0 [0–1]	
Washing item:		
5 + item 11 + item 17	1 [0–4]	
Obsessing:		
item6 + item 12 + item 18	4 [2–7]	
**Presence of clinically significant OC symptoms** **OCI-***R* ≥ **21**	N (%): 63 (41.7%)	
**CPAS**		
**Items**	**Median [IQR]**	
CPAS 1 Preoccupation with details	2 [1–3]	
CPAS 2 Perfectionism	2 [1–3]	
CPAS 3 Workaholism	1 [0–3]	
CPAS 4 Over-conscientiousness	3 [1–3]	
CPAS 5 Hoarding	1 [0–2]	
CPAS 6 Need for control	3 [1–4]	
CPAS 7 Miserliness	1 [0–2]	
CPAS 8 Rigidity	2 [1–3]	
**CPAS tot**	**14 [10–21]**	
**Presence of OCPD**	N (%): 56 (37)	
**Co-occurrence of clinically significant OC symptoms and OCPD**	38 (25)	

**Abbreviations**: CPAS: Compulsive Personality Assessment Scale; OCD: Obsessive Compulsive Disorder; OCI: Obsessive-Compulsive Inventory-revised; OCPD: Obsessive Compulsive Personality Disorder

The median total OCI-R score in the total sample was 17 [IQR: 10–28], with the highest subdomain scores in ordering (median 5 [IQR: 2–9]), obsessing (median 4 [IQR: 2–7]), checking (median 3 [IQR: 1–7]), suggesting these traits are particularly prominent in BMS patients, while Hoarding (median 1 [IQR: 0–5]) and Mental Neutralizing (median 0 [IQR: 0–1]) were less pronounced. Notably, 41.7% (n: 63) of patients met the criteria for having clinically significant OC symptoms (likely OCD) with an OCI-R total score 21.

The CPAS results further revealed pronounced OCPD traits in BMS patients, with a median total score of 14 [IQR: 10–21]. The highest median scores were for the items Need for Control (median 3 [IQR: 1–4]) and Over-Conscientiousness (median 3 [IQR: 1–3]), underscoring rigid, perfectionistic, checking and controlling tendencies. Lower scores were seen in domains such as Workaholism (median 1 [IQR: 0–3]) and Hoarding (median 1 [IQR: 0–2]). OCPD was present in 37% (n: 56) of the patients, e.g., *n* = 56 patients met the criteria for Obsessive-Compulsive Personality Disorder scoring 3 or higher on at least four of the eight items of the CPAS (DSM-5 diagnostic criteria) [52].

Interestingly, the co-occurrence clinically relevant OC symptoms and OCPD was found in 38 (25%) BMS patients highlighting a notable co-occurrence of these disorders in the BMS population. Specifically in 30 (20%) females and in 8 (5%) males.

Table 4 presents an analysis of the sociodemographic in two groups of BMS patients, categorized by their total OCI-R score (< 21 and **≥** 21). Notably, patients with clinically significant OC symptoms (OCI-R **≥** 21) exhibited a significantly higher BMI compared to those without (27.3 ± 4.12 vs. 25.7 ± 4.25, *p* = 0.030*). In contrast, systemic comorbidities and ACCI scores were generally comparable between the two groups. Additionally, Table 4 evaluates the sociodemographic characteristics between BMS patients with and without OCPD. Across sociodemographic clinical parameters no significant differences emerged between the groups; in terms of systemic comorbidities, both groups exhibited a high prevalence.

**Table 4:** Sociodemographic characteristics, systemic comorbidities, and drug consumption in two groups of BMS patients (OCI-*R* < 21 vs. OCI-*R* ≥ 21; without OCPD vs. with OCPD)

Sociodemographic characteristics	OCI -*R* Total Score < 21 (*N* = 88) No clinically significant OC symptoms	OCI -*R* Total Score ≥ 21 (*N* = 63) + clinically significant OC symptoms		Patients without OCPD (*N* = 95)	Patients with OCPD (*N* = 56)	
Gender	Frequency (%)	Frequency (%)	*P*-value	Frequency (%)	Frequency (%)	*P*-value
Female	73 (83)	50 (79.4)	0.728	79 (83.2)	44 (78.6)	0.629
Male	15 (17)	13 (20.60)		16 (16.8)	12 (21.4)	
	**Mean ± SD**	**Mean ± SD**		**Mean ± SD**	**Mean ± SD**	**P-value**
Age	62.8 ± 12.2	63.8 ± 12.3	0.595	61.1 ± 12.4	65 ± 11.8	0.169
Education (in years)	10.5 ± 4.2	9.38 ± 3.9	0.112	10.4 ± 4.1	9.32 ± 4.0	0.114
BMI	25.7 ± 4.3	27.3 ± 4.1	0.030*	26.1 ± 4.1	26.8 ± 4.5	0.317
***Family situation***	***Frequency (%)***	***Frequency (%)***	***P-value***	***Frequency (%)***	***Frequency (%)***	***P-value***
Married	57 (64.8)	57 (64.8)	0.228	60 (63.2)	46 (82.1)	0.108
Single	9 (10.2)	9 (10.2)		10 (10.5)	3 (5.4)	
Divorced	12 (13.6)	12 (13.6)		11 (11.6)	4 (7.1)	
Widowed	10 (11.4)	10 (11.4)		14 (14.7)	3 (5.4)	
***Employment statuts***	***Frequency (%)***	***Frequency (%)***	***P-value***	***Frequency (%)***	***Frequency (%)***	***P-value***
Employed	28 (31.8)	15 (23.8)	0.551	26 (27.4)	17 (30.4)	0.845
Unemployed	6 (6.8)	6 (9.5)		9 (9.5)	3 (5.4)	
Housewife	26 (29.5)	24 (38.1)		32 (33.7)	18 (32.1)	
Reteired	6(6.8)	18(28.6)		28 (29.5)	18 (32.1)	
***Smoke status***	***Frequency (%)***	***Frequency (%)***	***P-value***	***Frequency (%)***	***Frequency (%)***	***P-value***
Very Light smokers (< 5 cigarettes)	1 (1.1)	6 (9.5)	0.076	2 (2.1)	5 (8.9)	0.336
Light smokers (5–10 cigarettes)	7 (8)	2 (3.2)		6 (6.3)	3 (5.4)	
Moderate smokers (10–15 cigarettes)	11 (12.5)	6 (9.5)		12 (12.6)	5 (8.9)	
Heavy smokers (> 15 cigarettes)	7 (8)	4 (6.3)		7 (7.4)	4 (7.1)	
Non-smoker	62 (70.5)	45 (71.4)		68 (71.6)	39 (69.6)	
E-cig	0 (0)	3 (4.8)	0.071	0 (0)	3 (5.4)	0.049
Heat-not-burn (HNB) tobacco products	3 (3.4)	1 (1.6)	0.641	3 (3.2)	1 (1.8)	1.000
***Alcohol use***	***Frequency (%)***	***Frequency (%)***	***P-value***	***Frequency (%)***	***Frequency (%)***	***P-value***
Alcohol 1–2 units	6 (6.8)	5 (7.9)	0.738	9 (9.5)	2 (3.6)	0.222
Alcohol 2–3 units	2 (2.3)	4 (6.3)		3 (3.2)	3 (5.4)	
Alcohol >3 units	3 (3.4)	2 (3.2)		2 (2.1)	3 (5.4)	
No alcohol	77 (87.5)	52 (82.5)		81 (85.3)	48 (85.7)	
***Systemic Comorbidities***	***Frequency (%)***	***Frequency (%)***	***P-value***	***Frequency (%)***	***Frequency (%)***	***P-value***
Yes	80 (90.9)	54 (85.7)	0.434	86 (90.5)	48 (85.7)	0.428
No	8 (9.1)	9 (14.3)		9 (9.5)	8 (14.3)	
	***Median [IQR]***	***Median [IQR]***	***P-value***	***Median [IQR]***	***Median [IQR]***	***P-value***
Age-Adjusted Charlson Comorbidity Index (AACCI)	2 [1–3]	2 [1–3]	0.134	2 [1–3]	2 [1–3]	0.134
***Drugs Consumption***	***Frequency (%)***	***Frequency (%)***	***P-value***	***Frequency (%)***	***Frequency (%)***	***P-value***
Yes	74 (84.1)	49 (77.8)	0.283	80 (84.2)	43 (76.8)	0.283
No	14 (15.9)	14 (22.2)		15 (15.8)	13 (23.2)	

The significant difference between means was measured by the Student t-test

*Significance 0.01 < *p* ≤ 0.05. **Significance *p* ≤ 0.01

The significant difference between percentages was measured by the Pearson chi-square test (risk factors)

*Significance 0.01 < *p* ≤ 0.05. **Significance *p* ≤ 0.01

The significant difference between the percentages was measured by Fisher’s exact test (systemic comorbidities and drugs consumption)

**Significant with Bonferroni correction 0.001 (systemic comorbidities) [Patients with OCI-*R* < 21 vs. Patients with OCI-*R* ≥ 21)

**Significant with Bonferroni correction 0.002 (systemic comorbidities) [Patients without OCPD vs. Patients with OCPD)

**Significant with Bonferroni correction 0.003 (drugs consumption)

The significant difference between medians was measured by the Mann–Whitney U test

*Significance 0.01 < *p* ≤ 0.05. **Significance *p* ≤ 0.01

**Abbreviations**: ACCI: Age-Adjusted Charlson Comorbidity Index BMS: Burning Mouth Syndrome; BMI: Body Mass Index; O OCI-R: Obsessive-Compulsive Inventory Revised; OCPD: Obsessive Compulsive Personality Disorder

Table 5 compares the symptoms, pain levels, and neuropsychological profiles of BMS patients, grouped by their OCI-R scores [BMS patients without clinically significant OC symptoms (OCI < 21) versus BMS patients with clinically significant OC symptoms (OCI **≥** 21)]. No significant differences were found in referred oral symptoms, location pain intensity, and quality and patterns of symptomatology between the two groups.

**Table 5:** Symptoms, location and pain, analysis of neuropsychological profile, OCI items in two groups of BMS patients (OCI < 21 vs. OCI > 21; without OCPD vs. with OCPD)

Symptoms	OCI -*R* Total Score < 21 (*N* = 88) No clinically significant OC symptoms	OCI -*R* Total Score ≥ 21 (*N* = 63) + clinically significant OC symptoms		Patients without OCPD (*N* = 95)	Patients with OCPD (*N* = 56)	
	Frequency (%)	Frequency (%)	*P*-value	Frequency (%)	Frequency (%)	*p*-value
Burning	88 (100)	63 (100)	1.000	95 (100)	56 (100)	1.000
Xerostomia	62 (70.5)	44 (69.8)	1.000	65 (68.4)	41 (73.2)	0.584
Dysgeusia	41 (46.6)	34 (54)	0.412	45 (47.4)	30 (53.6)	0.503
Globus Pharyngeus	34 (38.6)	27 (42.9)	0.618	40 (92.1)	21 (37.5)	0.610
Subjective change in Tongue Morphology	33 (37.5)	23 (36.5)	1.000	37 (38.9)	19 (33.9)	0.603
Sialorrhea	17 (19.3)	14 (22.2)	0.687	20 (21.1)	11 (19.6)	1.000
Tingling Sensation	13 (14.8)	8 (12.7)	0.814	16 (16.8)	5 (8.9)	0.262
Intraoral Foreign Body Sensation	12 (13.6)	8 (12.7)	1.000	9 (9.5)	11 (19.6)	0.086
Itching	6 (6.8)	6 (9.5)	0.557	7 (7.4)	5 (8.9)	0.762
Oral Dyskinesia	4 (4.5)	7 (11.1)	0.202	5 (5.3)	6 (10.7)	0.331
Occlusal Dysesthesia	5 (5.7)	5 (7.9)	0.742	8 (8.4)	2 (3.6)	0.324
Subjective Halitosis	4 (4.5)	2 (3.2)	1.000	5 (5.3)	1 (9.8)	0.413
Dysosmia	0 (0)	2 (3.2)	0.172	2 (2.1)	0 (0)	0.530
***Location of pain/ Burning***	***Frequency (%)***	***Frequency (%)***	***P-value***	***Frequency (%)***	***Frequency (%)***	***p-value***
Tongue	85 (96.6)	62 (98.4)	0.641	91 (95.8)	56 (100)	0.297
Palate	58 (65.9)	44 (69.8)	0.725	64 (67.4)	38 (67.9)	1.000
Lips	56 (63.6)	43 (68.3)	0.605	59 (62.1)	40 (71.4)	0.289
Gums	51 (58)	39 (61.9)	0.737	57 (60)	33 (58.9)	1.000
Cheeks	51 (58)	36 (57.1)	1.000	55 (57.9)	32 (57.1)	1.000
Floor Of The Mouth	47 (53.4)	36 (57.1)	0.741	49 (51.6)	34 (60.7)	0.312
Trigone	45 (51.1)	32 (50.8)	1.000	48 (50.5)	29 (51.8)	1.000
***Pain***	***Median [IQR]***	***Median [IQR]***	***P-value***	***Median [IQR]***	***Median [IQR]***	***p-value***
VAS	10 [8–10]	9 [8–10]	0.185	10 [8–10]	10 [8–10]	0.890
T-PRI	12 [8–17]	14 [9.5–20]	0.134	12 [7-17.5]	14 [9-19.25]	0.209
***Symptoms pattern***	***Frequency (%)***	***Frequency (%)***	***P-value***	***Frequency (%)***	***Frequency (%)***	***p-value***
Worse In The Morning	4 (4.5)	3 (4.8)	1.000	4 (4.2)	3 (5.4)	0.711
Worse In The Evening	28 (43.2)	30 (47.6)	0.621	39 (41.1)	29 (51.8)	0.237
No Difference Between Morning and Evening	41 (46.6)	25 (39.7)	0.411	43 (45.3)	23 (41.1)	0.734
Continuous	38 (43.2)	30 (47.6)	0.621	39 (41.1)	29 (51.8)	0.237
Intermittent	43 (48.9)	27 (42.9)	0.510	49 (51.6)	21 (37.5)	0.128
Improve When Eating	26 (29.5)	25 (39.7)	0.224	27 (28.4)	24 (42.9)	0.077
***Neuropsychological Profile***	***Median [IQR]***	***Median [IQR]***	***P-value***	***Median [IQR]***	***Median [IQR]***	***P-value***
Total score of HAM-A	16 [14–20]	18 [15–21]	0.344	16 [14–20]	18 [15–21]	0.065
Total score of HAM-D	17 [13.75-20]	18 [15-21.5]	0.313	17 [12–20]	18 [15–23]	0.013
PSQI	8 [6–10]	9 [7.5–10]	0.047	8 [6–10]	9 [8–11]	0.004**
ESS	5 [3–6]	5 [4–7]	0.118	5 [3–6]	5.5 [4–7]	0.076
Sleep duration (in hours)	6 [5–7]	6 [5–7]	0.168	6 [5–7]	6 [5–7]	0.320
CGI-S	4 [4–5]	5 [4–5]	0.384	4 [4–5]	5 [4–5]	0.134
	**Frequency (%)**	**Frequency (%)**		**Frequency (%)**	**Frequency (%)**	
Insomnia onset prior to BMS diagnosis	53 (60.2)	44 (69.8)	0.234	55 (57.9)	42 (75)	0.037
History of previous mood disorder	48 (54.5)	31 (49.2)	0.620	47 (49.5)	32 (57.1)	0.402

The significant difference between the percentages of the symptoms and location was measured by Fisher’s exact test

**Significant with Bonferroni correction 0.003 (symptoms)

**Significant with Bonferroni correction 0.006 (location)

**Significant with Bonferroni correction 0.008 (symptoms pattern)

The significant difference between medians was measured by the Mann–Whitney U test

**Significant with Bonferroni correction 0.005

The significant difference between medians of the neuropsychological profile was measured by the Mann–Whitney U test

**Significant with Bonferroni correction 0.005

The significant difference between percentages was measured by the Pearson chi-square test

*Significance 0 01 < *p* ≤ 0 05. **Significance *p* ≤ 0 01

The significant difference between medians for OCI items was measured by the Mann–Whitney U test

**Significant with Bonferroni correction 0.002

The significant difference between medians of CPAS items was measured by the Mann–Whitney U test

**Significant with Bonferroni correction 0.006

The significant difference between percentages was measured by the Pearson chi-square test

*Significance 0 01 < *p* ≤ 0 05. **Significance *p* ≤ 0 01

**Abbreviations**: BMS: Burning Mouth Syndrome; CGI-S: Clinical Global Impression Severity of Illness; ESS: Epworth Sleepiness Scale; HAM-A: Hamilton Rating Scales for Anxiety; HAM-D: Hamilton Rating Scales for Depression; OCI-R: Obsessive-Compulsive Inventory revised; PSQI: Pittsburgh Sleep Quality Index, T-PRI: Total Pain Rating Index; VAS: Visual Analogue Scale; CPAS: Compulsive Personality Assessment Scale

In terms of neuropsychological profiles, HAM-A and HAM-D scores, did not significantly differ between the two groups. Additionally, despite BMS patients with clinically significant OC symptoms showed a trend toward worse outcomes in sleep quality, as indicated by higher PSQI scores (median 9 [7.5–10] vs. 8 [6–10], *p* = 0.047) compared with BMS patients without clinically relevant OC symptoms; this difference was not statistically significant.

Moreover, a higher percentage of BMS patients in significant OC symptoms group reported insomnia onset before BMS diagnosis (69.8% vs. 60.2%); however, this difference was also not statistically significant. Furthermore, Table 5 matches the symptoms, pain levels, and neuropsychological profiles of BMS patients with and without OCPD. No statistically significant differences were found in oral symptoms, as well as the location and pattern of symptomatology between the two groups.

Patients with OCPD had higher scores on the HAM-D (Median [IQR]: 18 [15–23] versus 17 [12–20]) (*p* = 0.013), indicating trends toward depressive symptoms and greater psychological distress. Additionally, the PSQI was significantly higher in the OCPD group (median 9 [8–11] vs. 8 [6–10], *p* = 0.004), suggesting that BMS patients with OCPD experience significantly worse sleep quality compared to those without OCPD.

Therefore, no statistically significant differences were detected in pain intensity and quality and in neuropsychological profiles between individuals with clinically significant OC symptoms and those without, neither in those with OCPD versus those without (apart from a trend indicating higher risk for developing depressive symptoms and a statistically significant difference in sleep quality in people with BMS and OCPD, when compared to those with BMS but without OCPD). The absence of other statistically significant differences could be due to the limited size of our sample.

## Discussion

This study is the first to examine the frequency and characteristics of OC symptoms and OCPD traits in patients with BMS, providing novel insights into the psychosomatic aspects of this chronic pain disorder. The findings revealed that 41.7% of the cohort were characterized by clinically significant OC symptoms, while 37% displayed Obsessive-Compulsive Personality Disorder, suggesting that the dimension of compulsivity is a relevant area to explore in BMS patients. These features add complexity to patients’ neuropsychological profiles, often complicating pain management and impacting treatment outcomes. In respect to the OCI-R scores, descriptive comparison was made between the BMS sample (*N* = 151) and an Italian normative sample (*N* = 340). It is important to emphasize that no statistical tests were conducted for this comparison due to the lack of individual-level data from the Italian normative sample. The data for the Italian normative sample were obtained from Marchetti et al. [48], with means and standard deviations summarized. Therefore, this comparison is purely descriptive and should be interpreted with caution to avoid potential bias. For this reason, the mean and standard deviation of the OCI-R scale were also measured to facilitate the comparison between these groups. Descriptive findings highlighted that BMS patients show higher scores on the total OCI-R scale and across all subscales. Specifically, the mean total OCI-R score for the BMS group was 20.3 ± 13.6, markedly higher than the Italian normative score of 7.8 ± 7.6. BMS patients had also significantly greater OCI-R scores across all subscales, with particularly high values on the ordering (5.5 ± 3.9 vs. 1.9 ± 2.3), obsessing (4.5 ± 3.5) and checking (4.1 ± 3.86 vs. 1.3 ± 2.0) dimensions. The larger standard deviations observed in the BMS cohort indicate greater variability in symptom severity within this group compared to the normative sample. For a detailed presentation of the summary data, including means and standard deviations, refer to **Supplementary File.** These findings are consistent with the literature on other chronic pain conditions, such as fibromyalgia and temporomandibular joint disorder, where rigid and perfectionistic behaviors similarly exacerbate both pain perception and pain management challenges [26, 47–50]. The results emphasize the importance of addressing this issue in BMS, as OC symptoms are likely to contribute to the overall burden of the disorder [55]. In our study, patients with BMS exhibited elevated scores on specific OCI-R subscales—such as ordering, obsessing, and checking—highlighting that these patients have a pronounced need for control and a tendency toward repetitive, compulsive behaviors. For instance, the ordering subscale score (median: 5) reflects an inflexible preoccupation with order, symmetry and control, which may lead to worrying about the asymmetrical oral areas that are involved in the painful sensations and maladaptive coping strategies like ritualistic behaviors that intensify stress rather than relieve it. The unpredictable and erratic nature of BMS symptoms compounds this issue, creating a vicious cycle in which the patients attempt to exert control over their pain, leading to increased distress and discomfort [56]. The dimension of obsessing, with a median score of 4, was another prominent factor. BMS patients frequently overthink, ruminate on their symptoms and engage in repetitive catastrophic thinking—a cognitive pattern closely linked to the worsening of chronic pain [57]. These intrusive, recurrent thoughts can increase the perceived intensity of pain, undermine coping strategies, prevent individuals from using relaxation techniques and hinder effective treatment [58]. Patients with such obsessive thoughts can find it difficult to comply with long-term therapy, as they may fixate on worst-case scenarios or fear treatment failure, leading to non-adherence or excessive reassurance-seeking [59]. Checking behaviors, with a median score of 3, often manifest as repetitive actions, such as frequent examinations of the mouth. Patients with the habit to check compulsively may monitor their symptoms excessively, look themselves in the mirror or be on hyperfocus and “scan” every perceived bodily sensation, which can positive reinforce this thinking loop and increase pain and anxiety in a vicious cycle [55, 56]. The persistent need for reassurance and symptom monitoring can become a significant obstacle in pain management: by fixating on minor painful sensations or inconsistencies in their symptoms, patients risk becoming frustrated and overwhelmed, and symptomatology may exacerbate. These symptoms define a particular cognitive profile characterized by rigidity and resistance to changes; one barrier to effective treatment could be the fear and anxiety related to change and the beginning of new form of treatments. Compulsive behaviours have a specific purpose in regulating emotions and reducing anxiety in everyday life, so patients might be afraid of therapy due to the fact that they would have to change their behaviour and possibly experience more anxiety. With regards to this latter point, there may be similarities with addictive disorders, in which individuals might be afraid of experiencing withdrawal.

The CPAS results further revealed pronounced presence of OCPD in BMS patients, with a median total score of 14 [IQR: 10–21], numerically higher than the mean CPAS score found in healthy subjects (3.7, SD: 3.1) [28]. Furthermore, scores from the CPAS items revealed that BMS patients had elevated measures of need for control (median = 3), over-conscientiousness (median = 3), rigidity (median = 2), and perfectionism (median = 2), which often translate into inflexible approaches to pain management. These traits lead patients to develop rigid thinking patterns that, while intended to mitigate symptoms, may paradoxically increase stress and exacerbate their condition. Rigidity could complicate pain management, as patients with inflexible expectations could struggle with treatment adherence and feel heightened anxiety when therapeutic plans deviate from their anticipated structure or fail to produce immediate results. These patients might not notice gradual improvement with therapy, as they are incline to an “all or nothing” way of thinking, and even the slightest residual symptom can be perceived as treatment failure and persistence of the condition, which becomes the center of their inner world [60, 61].

Despite the high levels of OC symptoms and OCPD traits, no significant differences were observed in pain severity or quality, as measured by the VAS and T-PRI, between BMS patients with and without these traits. This may be due to the high baseline pain levels in BMS patients, which could obscure the impact of obsessive-compulsive traits on pain modulation. However, the presence of these traits may still have significant long-term implications, contributing to increased stress, emotional dysregulation, and poorer treatment outcomes over time [62].

Patients with OCPD are depicted by great cognitive rigidity. Cognitive rigidity may serve as a technique to mitigate the pathological uncertainty or doubt and is believed to adversely affect the therapeutic efficacy of cognitive behavioural treatment (CBT), while its influence on the responsiveness to pharmacological therapy and in particular selective-serotonin reuptake inhibitors (SSRIs) remains ambiguous [63, 64]. Research indicates that individuals who exhibit rigidity and obstinacy tend to be more resistant to treatment overall [65, 66] and face an elevated chance of symptomatic recurrence. A naturalistic 5-year prospective follow-up study of individuals with OCD revealed that participants with concomitant OCPD were almost twice as likely to have relapse (*p* < 0.005) [35].

Similarly, no significant differences were found in anxiety and depression scores, likely due to the already high levels of psychological distress across all BMS patients, leading to a “ceiling effect” where additional obsessive-compulsive traits do not further elevate these scores [67].

We should point out a trend for higher risk of developing depressive symptoms in patients with BMS + OCPD, when compared to individuals with BMS but without OPCD; this could signify that the negative emotions linked to chronic distress caused by perceived oral pain might further evolve into mood problems, which could warrant psychiatric attention. The fact that OCPD constitutes an independent risk factor for developing depressive symptoms was thoroughly covered and emphasized by previous research [68]. Another important finding is the difference in sleep quality as measured in patients with OCPD compared to patients without OCPD. This might be due to rigid personality traits, including perfectionism and a strong need for control, that could exacerbate sleep disturbances by increasing the focus on the painful stimuli [69]. The finding is in line with previous research [70], where it was shown that sleep of those with features of OCPD was shorter, more disrupted, and characterized by lighter pattern. Given the established link between poor sleep and chronic pain, assessing and addressing sleep quality in BMS patients—particularly those with OCPD traits—should be a priority.

The dual presence of OCD and OCPD traits in 25% of BMS patients underscores the significant overlap between compulsive behaviors, rigid personality patterns, and perfectionistic thinking. This finding aligns with research in other chronic pain conditions, such as fibromyalgia and migraines, where higher rates of both disorders are similarly observed [27]. Patients with both OCD and OCPD may engage in repetitive, stress-inducing rituals while simultaneously resisting treatment flexibility, and maintaining strict control over their treatment [71]. This dual burden complicates pain management, as they may struggle to follow treatment plans consistently, resist necessary adjustments, experience heightened frustration when outcomes deviate from their expectations, and frequently develop mood problems or sleep disturbances.

Given the high prevalence of OCD and OCPD traits in BMS patients, their evaluation should go beyond standard assessments of anxiety, depression, and sleep disturbances. Traditional measures often miss the specific anxiety patterns and rigid personality traits—such as intrusive thoughts, compulsive behaviors, perfectionism, and a strong need for control—that can impact pain perception and generate poorer clinical outcomes.

This study suggests that targeted tools like the OCI-R and CPAS can provide valuable insights into how obsessive-compulsive tendencies and rigid thinking affect BMS patients’ ability to manage symptoms. These assessments are especially useful when patients display behaviors indicative of obsessive-compulsive patterns, such as frequently examining their mouths or fixating on minor changes in appearance. These tools can help clinicians better understand the neuropsychological factors contributing to symptom distress, enabling more tailored and effective treatment strategies.

A comprehensive approach that includes assessment for pain, obsessive-compulsive symptoms and traits, carried out by a multidisciplinary team (dentist, psychiatrist, psychologist, neurologist, etc.), alongside traditional evaluations of anxiety, depression, and sleep, provides a more holistic understanding of the BMS patient experience. This expanded evaluation is important to address both the physical symptoms and the underlying psychological difficulties that might influence recovery. Indeed, the identification of OC symptoms and OCPD traits allows clinicians to anticipate challenges with the ability to flexibly adapt to the therapeutic process by patients, resistance to gradual progresses and reluctance to accept adjustments in treatment plans.

Patients with BMS have been found to respond to pharmacological treatment with medications such as Vortioxetine [72]. In respect to the concomitant presence of clinically significant OC symptoms and likely diagnosis of OCD, as indicated by an OCI-R score equal to or above 21 in this group of patients, we know that cognitive-behavioral strategies, particularity with exposure and response prevention techniques, are effective in targeting compulsive behaviors [13]. Additionally, patients with pronounced obsessive-compulsive symptoms and traits may benefit from pharmacological adjustments, such as higher doses and longer durations of treatment with selective serotonin reuptake inhibitors (SSRIs) [13]. SSRIs show promising efficacy also in pain disorders [73], and could therefore offer a double benefit for this specific group of patients (i.e., reduce pain and ameliorate obsessive-compulsive symptoms). These treatment strategies would be of foremost importance if a formal diagnosis of OCD would be present. Moreover, we should point out that the presence of OCPD has been found to be a predictor of treatment resistance in patients with OCD [35]. Integrating targeted behavioral therapies with medical treatments is essential for managing complex chronic pain conditions influenced by obsessive-compulsive tendencies and cognitive vulnerabilities, ultimately leading to better pain management and improved quality of life for BMS patients.

### Limitations

This study has several limitations that should be considered when interpreting the findings. First, as a cross-sectional study, it provides a snapshot of the frequency and characteristics of OC symptoms and traits in BMS patients, but it cannot establish causality or examine changes over time. Longitudinal studies would be necessary to determine whether these traits contribute to the progression of BMS symptoms and poorer treatment outcome.

Second, reliance on self-reported measures (e.g., OCI-R and CPAS) may introduce response bias, and future research would benefit from incorporating structured clinical interviews.

Additionally, although this study included a relatively large cohort of BMS patients, the sample was limited to a single geographic area and most of participants were females, hence hindering generalizability. Further studies, with larger and more diverse samples and across different cultural backgrounds, are needed to corroborate our results.

## Conclusions

This study represents the first comprehensive analysis of the frequencies and characteristics of OC symptoms and OCPD traits among patients with BMS and provides new insights into the psychosomatic dimensions of this complex chronic pain disorder. The high frequency of clinically significant OC symptoms (that indicate likely diagnosis of OCD) (41.7%) and OCPD (37%) observed in the BMS cohort suggests that compulsive behaviors and rigid personality features are not only common but clinically relevant in this cohort, potentially complicating pain management, increasing stress, and reducing treatment adherence.

Furthermore, the overlap of clinically significant OC symptoms and OCPD in 25% of patients further highlights how compulsivity, rigidity, and perfectionism may drive maladaptive coping mechanisms, thereby limiting treatment flexibility and responsiveness.

These findings highlight the importance of a comprehensive, multidimensional approach to BMS, one that includes specific assessments for obsessive-compulsive symptoms and OCPD traits—using tools like the OCI-R and the CPAS—in addition to standard evaluations for anxiety, depression, and sleep disturbances.

Early recognition of OCD and OCPD would allow clinicians to better anticipate potential challenges related to treatment adherence and cognitive vulnerabilities, facilitating the development of targeted interventions. These may include cognitive-behavioral therapy to address compulsive behaviors and cognitive rigidity, as well as tailored pharmacologic adjustments. By integrating psychological and pharmacological strategies, healthcare providers may improve pain management outcomes, slow cognitive decline, and enhance the overall quality of life for BMS patients exhibiting obsessive-compulsive tendencies.

## Electronic supplementary material

Below is the link to the electronic supplementary material.

Supplementary Material 1

## Data Availability

No datasets were generated or analysed during the current study.
